# Real-life use of budesonide/formoterol in clinical practice: a 12-month follow-up assessment in a multi-national study of asthma patients established on single-inhaler maintenance and reliever therapy 

**DOI:** 10.5414/CP202224

**Published:** 2015-04-24

**Authors:** Björn Ställberg, Ian Naya, Jan Ekelund, Göran Eckerwall

**Affiliations:** 1 Uppsala University, Uppsala, Sweden,; 2 AstraZeneca, Macclesfield, UK,; 3 Scandinavian Surgical Outcomes Research Group, University of Gothenburg, Gothenburg, and; 4 AstraZeneca R&D, Mölndal, Sweden

**Keywords:** inhaled corticosteroid/long-acting β2-agonist, inhaler use, observational study, real-world evidence, treatment

## Abstract

Objective: The efficacy and safety of budesonide/formoterol maintenance and reliever therapy (MRT) has been demonstrated in phase III clinical studies, but limited data are available in a real-life setting. We examined the pattern of maintenance and as-needed inhaler use in routine clinical practice among patients with asthma receiving budesonide/formoterol MRT (NCT00505388). Methods: This 12-month European observational study enrolled patients prescribed budesonide/formoterol MRT and grouped them based on regimen: 80/4.5 µg one inhalation twice daily (b.i.d.); 160/4.5 µg one inhalation b.i.d.; 160/4.5 µg two inhalations b.i.d. (all plus as needed). Patient data were collected daily using an interactive voice- or web-response system. The primary outcome measure was total number of budesonide/formoterol inhalations/day. Results: Overall, 4,581 patients were included (64% female; mean age 48.4 years; regimen: 80/4.5 µg, n = 119; 160/4.5 µg, n = 3,106; 2 × 160/4.5 µg, n = 1,355). Mean (median) total numbers of budesonide/formoterol inhalations/day were 2.48 (2.11), 2.53 (2.14), and 4.27 (4.05) for 80/4.5 µg b.i.d., 160/4.5 µg b.i.d., and 2 × 160/4.5 µg b.i.d., respectively; corresponding mean (median) number of as-needed inhalations/day were 0.68 (0.17), 0.73 (0.26), and 1.08 (0.45), respectively. As-needed budesonide/formoterol use was generally low with a mean of 61 – 66% of reliever-free days; over 4 reliever inhalations/day occurred on a mean of 0.4 – 2.5% of days for all budesonide/formoterol MRT regimens. Conclusions: In routine clinical practice, all budesonide/formoterol MRT regimens were associated with a high proportion of reliever-free days and low incidence of high reliever-use days, indicating acceptable levels of asthma control with this symptom-adjusted controller regimen.

## Introduction 

Asthma is a chronic inflammatory airway disease that places a large burden both on patients and society [1]. It is characterized by variable and recurring symptoms, reversible airflow obstruction, and bronchospasm, which can lead to lung impairment, sleep disturbances, and limitations of daily activity [1]. 

The main goal of asthma management is to control and prevent symptoms and exacerbations in order to achieve optimal lung function and quality of life [1, 2]. This is normally achieved through the long-term use of both maintenance (long-term anti-inflammatory) and reliever (short-term symptom relief) therapies. Current recommendations suggest that patients with asthma of at least moderate severity are treated with maintenance inhaled corticosteroid/long-acting β_2_-agonist (ICS/LABA) therapy plus a rapid-acting bronchodilator as reliever [1]. However, in a large number of countries (and throughout Europe) fixed-dose budesonide/formoterol is available as a maintenance treatment with as-needed adjustments taken as reliever therapy when symptoms occur. 

A growing body of evidence from over 14,000 patients from double-blind phase IIIa/IIIb studies indicates the improved efficacy and safety of budesonide/formoterol maintenance and reliever therapy (MRT) compared with traditional fixed-dose maintenance and traditional short-acting β_2_-agonist (SABA) reliever therapy [3, 4, 5, 6, 7, 8]. Budesonide/formoterol MRT was shown to provide effective asthma control and reduce the number and risk of severe exacerbations compared with other ICS/LABA combinations at similar or higher maintenance doses [3, 4, 5, 6]. This novel regimen has also improved overall asthma control at a lower corticosteroid load compared with conventional best practice (including ICS/LABA plus SABA therapy at the physician’s discretion, as well as any additional controller medications at Global Initiative for Asthma [GINA] steps 3 and 4) [9, 10, 11]. 

As-needed reliever use is recorded solely as median SABA use in many randomized clinical trials with fixed-dose regimens, giving little information for the 50% of patients with asthma that is not well controlled [12, 13, 14]. Clinical trials of budesonide/formoterol MRT have shown mean use of as-needed medication to be approximately one inhalation/day in patients with uncontrolled asthma on conventional GINA step 2 – 4 therapy [3, 4, 5, 6, 7, 8], but it is not known how usage patterns of MRT translate to clinical practice in a typical asthma population. Therefore, detailed knowledge on the levels of as-needed budesonide/formoterol use in real life is needed to accurately indicate the degree of asthma control and potential for over-use of this regimen [15]. These data will also be of use in gauging the true costs of implementing budesonide/formoterol MRT use in routine clinical practice. 

To examine the use of budesonide/formoterol MRT in real-life clinical practice amongst patients who had been prescribed the regimen, this observational study investigated the usage profile of budesonide/formoterol MRT in European clinical practice to establish if actual use was consistent with 1) the label, 2) target levels of as-needed medication use indicating good asthma control, and 3) published randomized prospective trial data on the product. Therefore, no efficacy outcome measures were examined or reported in this study. 

## Methods 

The pattern of maintenance and as-needed inhaler use among patients with asthma receiving an existing regimen of budesonide/formoterol MRT was examined during a 12-month, observational study in twelve European countries (Belgium, Bulgaria, Czech Republic, Denmark, Germany, Greece, Hungary, the Netherlands, Norway, Portugal, Sweden, and the United Kingdom; NCT00505388). This observational study was undertaken to fulfil regulatory commitments and examine budesonide/formoterol MRT in real-life clinical practice following the approval and introduction of budesonide/formoterol MRT in Europe. 

## Study design 

The study involved two planned visits to the clinic: one at baseline (inclusion) and one after 12 months. Between the two planned study visits, patients were treated and assessed in accordance with normal clinical practice (participation in the study did not change the asthma treatment they received). A 12-month study design/duration of assessment was planned to minimize the influence of seasonal inhaler use. 

Patient data were collected on a daily basis using an interactive voice-response system (IVRS) and/or interactive web-response system (IWRS; ICON Clinical Research, Dublin, Ireland). At inclusion, patients were instructed how to use IVRS/IWRS to record and report their medication usage daily during the study period. The frequency at which patients reported data was monitored (but actual data on adherence were not monitored on a regular basis), and patients were sent reminders if they did not use the system frequently or had poor adherence. 

## Patient population 

To be eligible for the study, patients were required to have received an asthma diagnosis and to have been prescribed budesonide/formoterol MRT prior to entry (so as to reflect actual real-world patient usage and not protocoled use of the regimen). Patients were also required to provide signed and dated informed consent. There were no exclusion criteria to limit patient characteristics. Patients with asthma were treated according to normal clinical practice and followed instruction according to the drug label. At inclusion, patients were segmented into one of three groups by their prescribed daily maintenance dose of budesonide/formoterol: 80/4.5 µg, one inhalation twice daily (b.i.d.); 160/4.5 µg, one inhalation b.i.d.; 160/4.5 µg, two inhalations b.i.d. (all plus budesonide/formoterol as-needed). Discontinuations were permitted at the discretion of the investigator. Minimal baseline data were collected using the IVRS system, which included gender, age, and race. 

## Outcome measures 

The primary outcome measure was the total number of IVRS/IWRS-reported budesonide/formoterol inhalations/day; secondary outcomes included the number of as-needed (reliever) inhalations with budesonide/formoterol. The number of maintenance inhalations was calculated as the difference between total and as-needed inhalations. 

## Statistical analyses 

Descriptive statistical analyses (number of observations, median, mean, standard deviation, minimum and maximum) were performed, and plots illustrating different aspects of the daily use of budesonide/formoterol were prepared. Since no statistical hypothesis test was planned, the sample size was not based on any formal power calculation but was based on the intention to describe budesonide/formoterol MRT as accurately as possible. Initially the study aimed to recruit 8,000 patients, but two interim analyses showed there was no evidence of misuse of the budesonide/formoterol MRT concept. Therefore, the target number was reduced to 4,400. The full analysis set was used in all descriptions of data and comprised all included patients with diary data after inclusion in the study. 

## Results 

### Patients 

**Figure 1.**
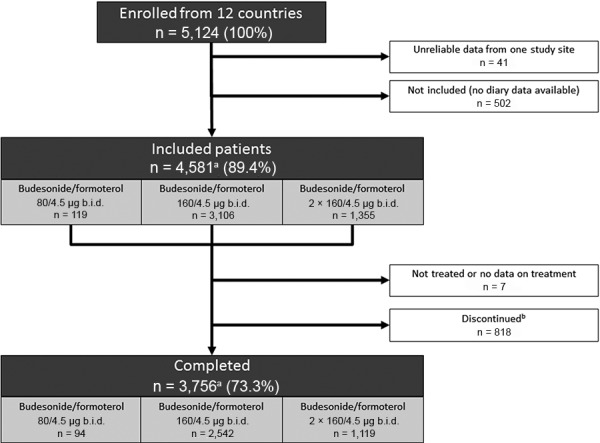
Patient disposition throughout the study. ^a^Includes 1 patient wrongly prescribed 320/18 µg who was only included in the “all patients” group; ^b^reasons for discontinuation include: incorrect enrolment (n = 34); terminated study treatment (n = 106); voluntary discontinuation by patient (n = 453); lost to follow-up (n = 134); and severe non-adherence to protocol (n = 91). b.i.d. = twice daily.

The first patient was enrolled in July 2007 and the last patient finished the study in April 2010. Of 5,124 patients enrolled from twelve European countries, diary data were available for 4,581 patients (89.4%; the full analysis set); 41 patients were excluded because one study site was regarded as unreliable and 502 were not included due to the absence of IVRS/IWRS diary data. From 4,581 included patients, 119 (3.0%), 3,106 (67.8%), and 1,355 (29.6%) were initiated on the budesonide/formoterol MRT regimen using a target maintenance level of 80/4.5 µg b.i.d., 160/4.5 µg b.i.d., and 2 × 160/4.5 µg b.i.d., respectively. In total, 818 patients discontinued treatment; reasons for discontinuation included voluntary discontinuation by subject (453 patients), patient unavailability (134 patients), termination of study treatment (106 patients), severe non-compliance with protocol (91 patients) or incorrect enrolment (34 patients). A further 7 patients were either untreated or had no data on treatment available. A total of 3,756 patients (73.3% of patients enrolled) completed the study; 94 patients in the 80/4.5 µg group, 2,542 patients in the 160/4.5 µg group and 1,119 patients in the 160/4.5 µg group (Figure 1). One patient was treated with an alternative regimen than recommended (two inhalations b.i.d. of 80/4.5 µg (320/18 µg maintenance)) and was only included in an “all patients” group. 

Patients’ baseline characteristics are described in Table 1. The mean age was 48.4 years (range 17 – 89), 64% were female, and the population was predominantly Caucasian. 

## Medication exposure 

### Total medication use 

The median exposure to budesonide/formoterol was 344 days, which was consistent across the three cohorts. Mean (median) days of exposure for the budesonide/formoterol 80/4.5 µg b.i.d., 160/4.5 µg b.i.d., and 2 × 160/4.5 µg b.i.d. groups were 267 (337), 269 (345), and 268 (342) days, respectively. 

**Figure 2.**
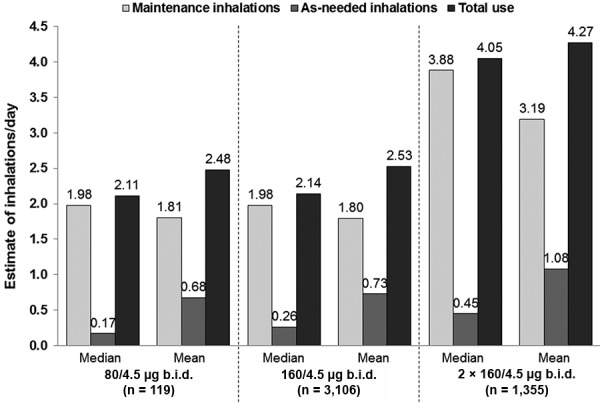
Median and mean number of inhalations/day (maintenance, as-needed, and total use) by treatment group. b.i.d. = twice daily.

Overall, the mean (median) numbers of budesonide/formoterol inhalations/day, including maintenance and as-needed use for the 80/4.5 µg b.i.d., 160/4.5 µg b.i.d., and 2 × 160/4.5 µg b.i.d. groups, were 2.48 (2.11), 2.53 (2.14), and 4.27 (4.05), respectively. Mean (median) inhalations/day for maintenance, as-needed, and total use are shown in Figure 2. The mean and median numbers of inhalations for each dose cohort by country are described in Table 2. In general, the median and mean numbers in the lower-dose groups were around 2.1 and 2.5 inhalations/day, but almost doubled in the higher-dose group due to the higher maintenance dose. Across all twelve countries, the level of total budesonide/formoterol inhalations/day for the most commonly used regimen (160/4.5 µg b.i.d. plus as-needed) showed a reduced variability for the median versus mean estimates of use by country; these values ranged between 2.0 – 2.2 and 2.1 – 2.9, respectively. 

**Figure 3.**
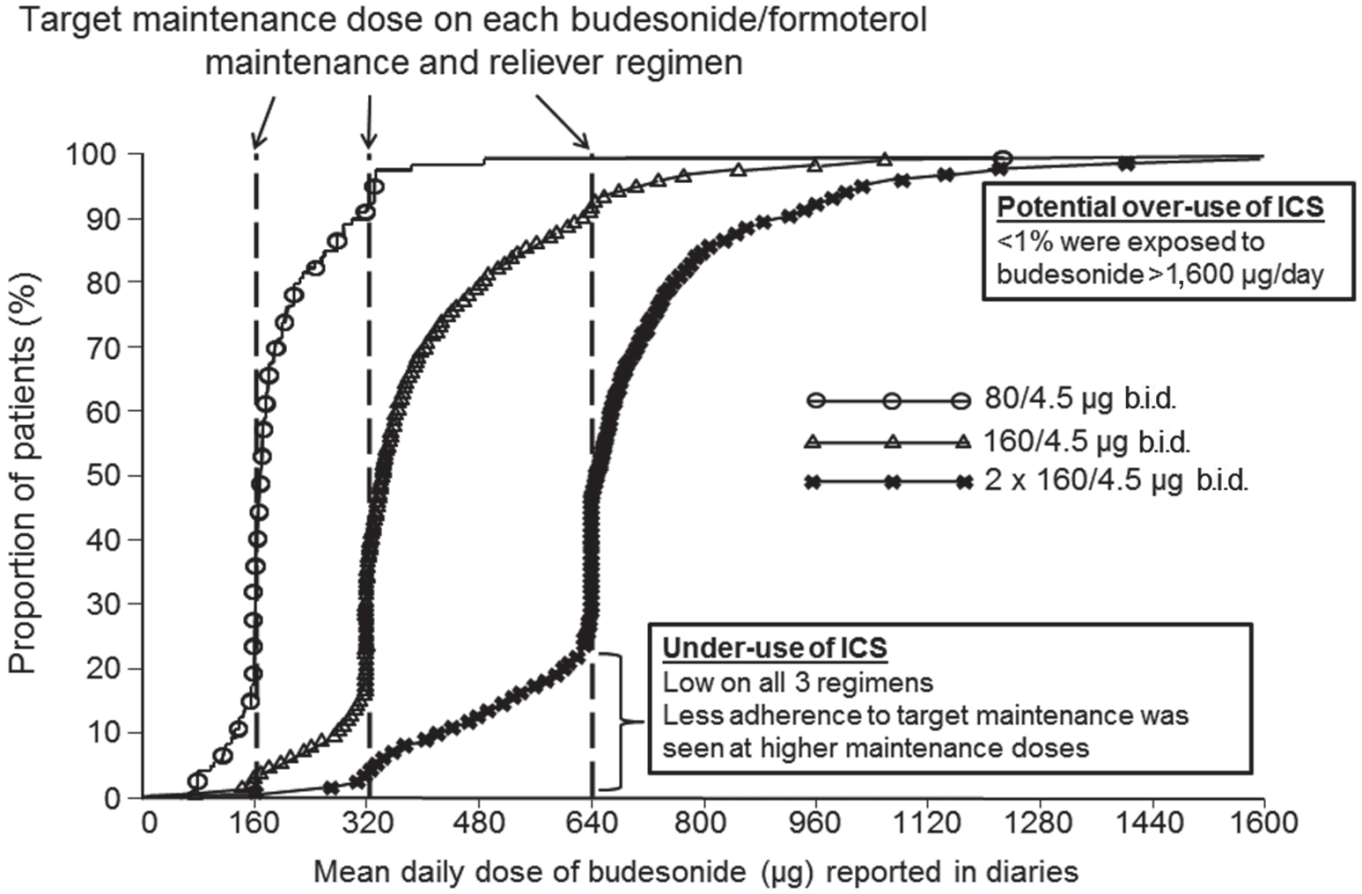
Cumulative range of total budesonide exposure (both maintenance and reliever use). b.i.d. = twice daily; ICS = inhaled corticosteroid.

There were only small proportions of patients with very low or very high budesonide use (Figure 3). The proportion of patients with potential over-use was very small and < 1% of patients were exposed to budesonide doses > 1,600 µg/day. Under-use was uncommon with all three regimens: < 5% of patients in each treatment group used less than one-half of the prescribed maintenance dose. 

### As-needed medication use 

As-needed use of budesonide/formoterol was generally low but higher in patients who received the higher maintenance dose (Figure 2). The mean (median) numbers of as-needed inhalations/day were 0.68 (0.17), 0.73 (0.26), and 1.08 (0.45) for 80/4.5 µg b.i.d., 160/4.5 µg b.i.d., and 2 × 160/4.5 µg b.i.d. groups, respectively. Median as-needed use was below two inhalations/week for 50% of patients taking two maintenance inhalations of budesonide/formoterol, and 50% of patients taking the highest recommended dosage (2 × 160/4.5 µg b.i.d. plus as needed) used a maximum of 3.1 inhalations/week. When pooling all data it was apparent that, overall, 59% of patients had a mean as-needed use of ≤ 0.5 inhalations/day (< 3.5 inhalations/week). 

**Figure 4.**
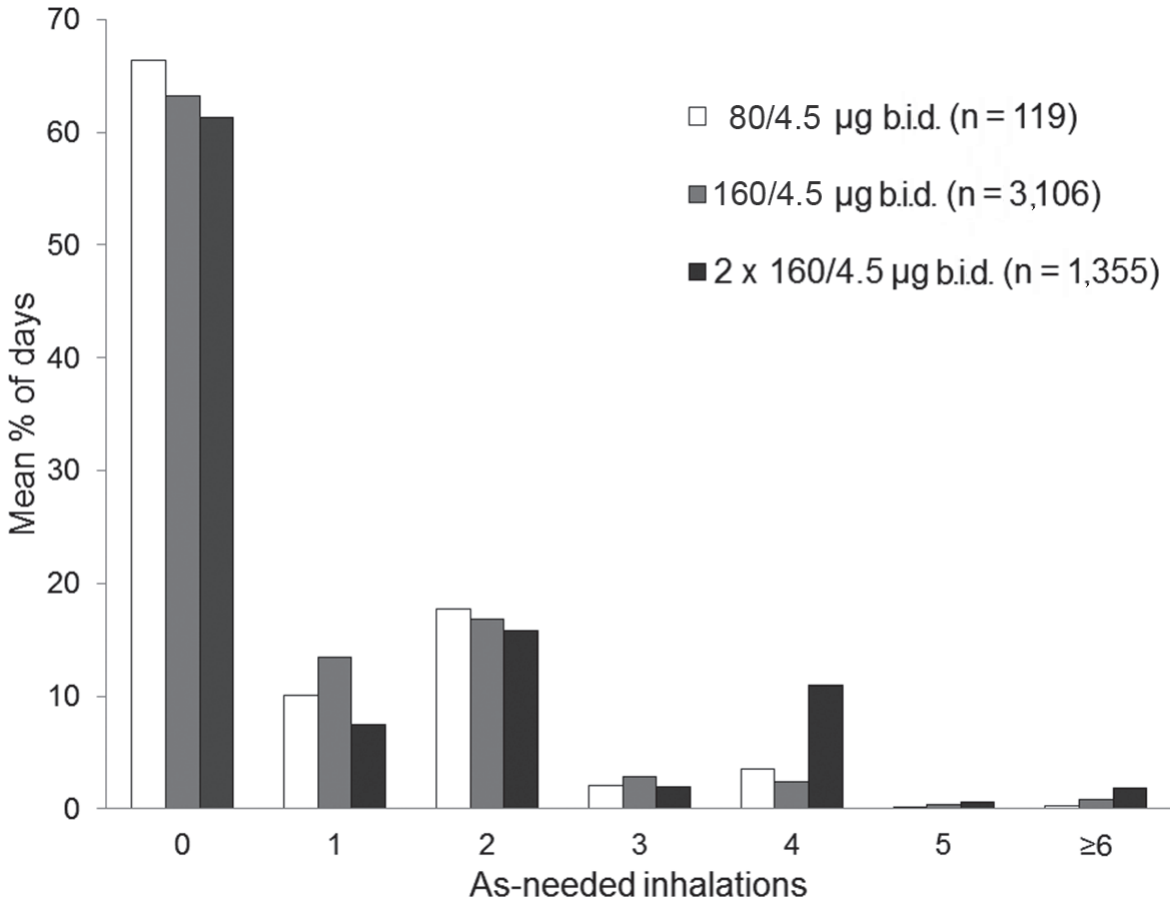
Mean percentage of days with different budesonide/formoterol as-needed inhalation use. b.i.d. = twice daily.

For ~ 2/3 of the study days, patients did not require any as-needed inhalations of budesonide/formoterol (Figure 4). Patients in the two lower-dose groups had slightly more reliever-free days and fewer high reliever-use days. Across all three treatment groups, high as-needed use (over four inhalations/day) was observed on 1.5 (0.4%), 4.3 (1.2%), and 9.0 (2.5%) days of the study period for the 80/4.5 µg b.i.d., 160/4.5 µg b.i.d., and 2 × 160/4.5 µg b.i.d. groups, respectively. 

### Maintenance use 

Maintenance use varied across all three regimens but was considered to be closer to physicians’ prescription targets in the two lower-dose budesonide/formoterol cohorts: patients in the 80/4.5 µg and 160/4.5 µg b.i.d. groups had a reported mean of 1.8 inhalations/day (versus a prescribed number of two), compared with 3.2 inhalations/day (versus a prescribed number of 4) for the 2 × 160/4.5 µg b.i.d. group (Figure 2). 

## Discussion 

Adherence to asthma medication is an important consideration during treatment as it is low irrespective of patient age [16, 17, 18], decreases further amongst those patients who present with difficult-to-control asthma [17, 19], and is correlated with negative outcomes [17, 18, 20]. Following the approval and introduction of the budesonide/formoterol MRT in Europe, this observational study was undertaken to fulfil regulatory commitments and examine this new treatment regimen in real-life clinical practice. 

### Main findings 

As-needed (reliever) use of budesonide/formoterol was low across all three regimens of budesonide/formoterol MRT. Furthermore, the high number of reliever-free days combined with the low incidence of high reliever-use days in all regimens suggests that all budesonide/formoterol MRT regimens were associated with appropriate levels of asthma control in the vast majority of patients when used in normal clinical practice. Overall, the three regimens were similar in terms of as-needed reliever use, but higher prescribed maintenance doses of budesonide/formoterol were associated with incremental increases in as-needed medication use. Thus, these data suggest that higher maintenance doses were not associated with greater levels of asthma control but likely reflect a marker of disease severity and/or worse compliance with regular ICS/LABA maintenance therapy. 

While the budesonide/formoterol MRT regimen could be perceived to increase the risk of ICS over-use, no signs of apparent misuse were evident in this large real-life study. Under- and over-use were low and, based on the present results, ~ 90% of patients were likely to have received a dose of ICS (~ 320 µg/day of budesonide) that is reported to provide 80% of the clinical benefit on current control during periods of stable asthma [21]. The absence of need for reliever therapy reported on all regimens in close to 2/3 of treatment days further corroborates these findings. These results are in agreement with those of Patel et al., who recently showed that the efficacy of budesonide/formoterol MRT was superior to fixed-dose budesonide/formoterol plus SABA when using electronic adherence trackers [15]. 

### Strengths and limitations of this study 

The strengths of this study include the large size of the study population and the geographic spread of included countries (twelve representative European countries where the regimen was first launched). The use of the IVRS/IWRS provided a patient-friendly and reliable means of collecting data, but, importantly, also provided a means of reminding patients who were not frequently reporting data, thereby ensuring consistency and thoroughness of the dataset. Additionally, the observational nature of the design combined with the absence of exclusion criteria relating to patient characteristics/comorbidities and freedom to co-prescribe other medications yielded a population of patients that can be considered to represent real-life clinical practice. This is an important consideration as patients in randomized controlled trials are not usually representative of clinical practice [22], and so observational studies such as this one are invaluable for informing policy makers and improving guidelines [23]. 

Potential weaknesses of the study include the lack of use of any objective measurements to determine exacerbation rates and asthma control or airway inflammation, as well as the absence of documentation of allergic status and concomitant asthma medication use. Such data would have enabled further conclusions to be drawn between treatment use and asthma symptoms and control. It should also be noted that asthma is a disease that may change in severity over time and, therefore, a study of longer duration would be required to more closely assess disease changes. The high degree of adherence observed in this study also needs to be considered. It is possible that the rate of adherence may have been influenced by use of the IVRS/IWRS system and its associated automatic reminder service, thus giving an inaccurate representation of real-life medication use. 

The overall discontinuation rate for this study was 17.9% (818 patients); of this number, 453 patients voluntarily discontinued treatment. The reasons for this are not clear as investigators were not asked to record why patients discontinued. 

Data for confounding factors such as concomitant medications (including reliever medication), level of patient education, smoking behavior and motivation for seeking treatment were not collected during this study. It is possible that collection of such data would enhance the findings of future studies and so we recommend that this information be gathered where possible. 

Patient age in this study ranged from 17 to 89 years, with a median age of 48.4 years. Given the expanded upper age range of this population, clinical diagnosis becomes more complex; it is possible that patients near the top of this range may have a mixed diagnosis of asthma and/or chronic obstructive pulmonary disease. Therefore, these patients may not provide results which accurately represent an asthmatic-only population. Future sub-analyses of treatment results by age might serve to clarify this point. 

### Interpretation of findings in relation to previously published work 

As-needed use of budesonide/formoterol was reduced in this study compared with randomized clinical trials, and the proportion of days with increased as-needed medication use was lower. The mean number of as-needed inhalations/day was ~ 1 in randomized clinical trials of uncontrolled patients on existing standard of care therapy [3, 4, 5, 6, 7, 8], compared with a mean of 0.7 inhalations/day in this study (on the most commonly used 160/4.5 b.i.d. regimen). The difference observed between randomized clinical trials and this study is likely to be a result of differences in the inclusion criteria and resulting patient population. For example, in the randomized trials, patients were required to have used a SABA on 4 out of 7 days of the baseline run-in period, thereby enriching the population to be high users of reliever therapy and potentially those with more severe disease. In contrast, no inclusion criteria were used in the present study. The results from this study are consistent with other prospective studies that employed less stringent inclusion criteria (with respect to baseline as-needed medication use) where mean as-needed reliever medication use was 0.6 – 0.9 inhalations/day [9, 24]. However, mean use alone fails to capture an accurate picture of reliever use and proportion of patients likely to achieve adequate control. Median values are more informative and were found to be substantially lower for all three regimens in this study. For the most commonly used budesonide/formoterol 160/4.5 µg b.i.d. MRT regimen, at least 50% of patients used less than two inhalations/week of reliever therapy and ~ 2/3 of days were reliever free. 

The current observational study indicates that the total use of budesonide/formoterol is lower in actual clinical practice than that seen in randomized clinical trials, suggesting that the MRT concept may be associated with lower treatment costs than expected. The average patient on the most commonly used 160/4.5 µg b.i.d. plus as-needed regimen had a mean of 2.1 – 2.9 total inhalations/day across all twelve countries. Based on clinical trial data, it is often assumed this regimen will require two inhalations of maintenance plus roughly one inhalation of as-needed medication (three inhalations/day) for all patients [3, 4, 6, 8], representing a 3 – 43% increase in mean medication cost relative to the findings reported here (for an average patient across the twelve different European countries). This discrepancy may be explained by higher levels of control and less as-needed use in the real-world setting compared with previous randomized clinical trials. Additionally, it may be due to the potential use of other controller medication, which is not allowed in randomized studies, or simply the presence of patients with less severe asthma. It also factors in the result of lower adherence to maintenance therapy. 

### Implications for future research, policy and practice 

The use of budesonide/formoterol MRT has been shown to be highly successful in asthma management in clinical trials compared with the current standard of care of a fixed-dose regimen. Furthermore, data from this observational study have demonstrated that this treatment regimen is employed successfully in everyday clinical practice outside the confines of a structured clinical study design, although in real-life reminders are not generally used and this may have positively influenced patient adherence. In order to ensure that patients received appropriate assessment and care throughout the study, two clinic visits were scheduled (at inclusion and 12 months later) and patients were treated and assessed according to normal clinical practice during the intervening period. This arrangement allowed any deterioration in asthma control to be identified and treated appropriately. Patients’ use of budesonide/formoterol MRT in routine clinical practice was consistent with the recommendations in the product label. 

## Conclusions 

In this real-life follow-up program across twelve countries, as-needed use of budesonide/formoterol as part of an MRT regimen was low. The high percentages of reliever-free days and low incidence of high reliever-use days indicate that acceptable levels of asthma control were achieved for most patients across all budesonide/formoterol MRT regimens in routine clinical practice. 

## Acknowledgments 

This study was funded by AstraZeneca. The sponsors were involved in the study design and interpretation of the data, always in conjunction with the study investigators. The authors would like to thank Matt Weitz, inScience Communications, Springer Healthcare for medical writing assistance in the preparation of this manuscript. This assistance was funded by AstraZeneca. Matt Weitz does not qualify as an author because he did not contribute to the conception or design of the study, the acquisition, analysis, or interpretation of data, nor did he provide final approval of the manuscript. 

## Conflicts of interest 

BS has received honoraria for educational activities from AstraZeneca, GlaxoSmithKline, Meda, Merck Sharp and Dohme, and has served on an advisory board arranged by AstraZeneca, Novartis, and Boehringer Ingelheim. 

GE is a full-time employee of AstraZeneca and holds stock in the company. 

IN was an employee of AstraZeneca at the time the analysis was conducted. 

JE is an ex-employee of AstraZeneca, but has no current conflicts to declare. 

**Table 1:** Patients’ baseline demographics.

Characteristic	Budesonide/formoterol			All patients (n = 4,581)
	80/4.5 µg b.i.d. (n = 119)	160/4.5 µg b.i.d. (n = 3,106)	2 × 160/4.5 µg b.i.d. (n = 1,355)	
Sex, no. (%)				
Male	42 (35)	1,099 (35)	490 (36)	1,631 (36)
Female	77 (65)	2,007 (65)	865 (64)	2,950 (64)^a^
Age, years				
Mean	46.0	47.8	50.1	48.4
Range	17 – 86	18 – 89	18 – 88	17 – 89

b.i.d. = twice daily. ^a^Includes 1 patient wrongly prescribed 320/18 µg who was only included in the “all patients” group.

**Table 2.** As-needed and total budesonide/formoterol use by country.

Country		As-needed doses		Maintenance + as-needed inh/day		
		inh/day	inh/week	80/4.5 µg b.i.d.	160/4.5 µg b.i.d.	2 x 160/4.5 µg b.i.d.
Belgium	No.	621		5	482	134
	Median	0.27	1.89	2.15	2.20	4.03
	Mean	0.86	6.02	2.27	2.56	4.17
Bulgaria	No.	125		2	96	27
	Median	0.25	1.75	2.07	2.14	3.32
	Mean	0.57	3.99	2.08	2.34	3.45
Czech Republic	No.	349		2	286	61
	Median	0.22	1.54	1.66	2.14	4.04
	Mean	0.80	5.60	1.66	2.54	4.15
Denmark	No.	68		5	45	18
	Median	0.20	1.40	2.09	2.12	4.03
	Mean	0.86	6.02	2.01	2.32	4.00
Germany	No.	465		18	370	77
	Median	0.35	2.45	2.07	2.10	4.00
	Mean	0.87	6.09	3.15	2.43	3.91
Greece	No.	562		7	258	297
	Median	0.27	1.89	2.08	2.20	4.00
	Mean	0.83	5.81	2.08	2.86	4.08
Hungary	No.	475		3	302	170
	Median	0.29	2.03	2.00	2.12	4.16
	Mean	0.81	5.67	1.94	2.45	4.48
Netherlands	No.	335		8	250	77
	Median	0.32	2.24	2.03	2.07	4.00
	Mean	0.77	5.36	2.09	2.49	3.95
Norway	No.	418		6	281	131
	Median	0.61	4.27	2.08	2.25	4.45
	Mean	1.11	7.77	2.36	2.72	4.78
Portugal	No.	282		3	254	25
	Median	0.04	0.28	1.76	2.00	3.81
	Mean	0.30	2.10	1.52	2.13	3.56
Sweden	No.	417		24	278	115
	Median	0.30	2.10	2.11	2.23	4.18
	Mean	0.67	4.69	2.40	2.53	4.37
United Kingdom	No.	464		36	204	223
	Median	0.58	4.06	2.43	2.20	4.18
	Mean	1.38	9.66	2.69	2.67	4.50
All	No.	4,581		119	3,106	1,355
	Median	0.30	2.10	2.11	2.14	4.05
	Mean	0.83	5.81	2.48	2.53	4.27

b.i.d. = twice daily; inh = inhalation.
